# Low vitamin D levels accelerates muscle mass loss in patients with chronic liver disease

**DOI:** 10.1371/journal.pone.0299313

**Published:** 2024-03-26

**Authors:** Tomomi Okubo, Masanori Atsukawa, Akihito Tsubota, Hiroki Ono, Tadamichi Kawano, Yuji Yoshida, Taeang Arai, Korenobou Hayama, Norio Itokawa, Chisa Kondo, Katsuhiko Iwakiri

**Affiliations:** 1 Department of Internal Medicine, Division of Gastroenterology, Nippon Medical School Chiba Hokusoh Hospital, Inzai, Japan; 2 Department of Internal Medicine, Division of Gastroenterology and Hepatology, Nippon Medical School, Tokyo, Japan; 3 Project Research Units, Research Center for Medical Science, The Jikei University School of Medicine, Tokyo, Japan; Gyeongsang National University, REPUBLIC OF KOREA

## Abstract

Sarcopenia frequently and progressively occurs in patients with chronic liver disease. This study aimed to clarify the relationship between vitamin D levels and muscle mass loss. A total of 166 patients with chronic liver disease were enrolled in this study. Skeletal muscle mass index (SMI) was measured by bioelectrical impedance analysis at baseline and after 1 year. The rate of change in SMI from baseline after 1 year was calculated: ΔSMI (%) = [(1-year SMI − baseline SMI) / baseline SMI] × 100. Muscle mass loss was defined as ΔSMI ≤ −2%. The median 25-hydroxyvitamin D was 15.2 (11.2–19.3) ng/mL. The median SMI were 6.8 (5.9–7.8) kg/m^2^ at baseline and 6.7 (5.9–7.6) kg/m^2^ after 1 year. The median ΔSMI was −1.23% (−2.21% to 1.61%). Multivariate analysis identified low 25-hydroxyvitamin D as an independent factor associated with muscle mass loss. The optimal cut-off value of 25-hydroxyvitamin D to predict muscle mass loss was 12.7 ng/mL. Muscle mass loss was found in 56.4% v.s. 18.0% of patients with 25-hydroxyvitamin D < 12.7 vs. ≥ 12.7 ng/mL, respectively (p = 9.01 × 10^−7^); with the highest incidence in patients with non-alcoholic fatty liver disease (NAFLD). Specifically, patients with NAFLD and 25-hydroxyvitamin D < 12.7 ng/mL had a significantly higher incidence of muscle mass loss than those with ≥ 12.7 ng/mL (p = 1.23 × 10^−3^). Low vitamin D levels are associated with muscle mass loss after 1 year in patients with chronic liver disease, especially NAFLD.

## Introduction

Sarcopenia is the loss of skeletal muscle mass and strength that commonly develops in patients with cirrhosis and can worsen prognosis [1, 2]. To diagnose sarcopenia, muscle mass and grip strength are measured based on the criteria published by several academic societies [3–5]. Importantly, not only sarcopenia but also muscle mass loss alone negatively impacts the prognosis in patients with cirrhosis [2]. Considering the association between muscle mass and clinical outcomes, it is crucial to measure muscle mass loss using some modalities [such as computed tomography (CT) imaging and bioelectrical impedance analysis (BIA)] and assess the prognosis in patients with cirrhosis [5–7]. In the general population, muscle mass decreases by approximately 1% per year from age 30 to 70 and 1.5% per year after age 70. Meanwhile, the annual rate of skeletal mass loss is 2.2% in patients with cirrhosis: 1.3%, 3.5%, and 6.1% in those with Child-Pugh class A, B, and C, respectively, indicating that it is considerably high and increases with disease progression [8].

Branched-chain amino acid (BCAA) and/or vitamin D supplementation and exercise can be effective in preventing liver disease-related sarcopenia [1, 9, 10]; however, there is no consensus on the optimal intervention strategy. The mechanism of sarcopenia is complex and multifactorial; in patients with cirrhosis, hyperammonemia, insulin resistance, use of loop diuretics, low testosterone, and low vitamin D are involved in its pathogenesis [11–15]. Several 3–5-year longitudinal studies of community-dwelling older adults have revealed that low vitamin D levels are associated with muscle mass loss and may contribute to the onset of sarcopenia [16–19]. Similarly, some cross-sectional studies have reported the relationship between low vitamin D levels and sarcopenia in chronic liver disease [15, 20]. In patients with decompensated cirrhosis, muscle mass loss is −3.3% and the incidence of sarcopenia naturally increases over a 1 year [10]. While these previous studies suggest the association of low vitamin D levels and muscle mass loss, it remains unclear whether low vitamin D levels affect muscle mass loss at the 1-year assessment in patients without decompensated cirrhosis. Patients with muscle mass loss at an earlier stage should be identified and their clinical characteristics determined to plan for appropriate early therapeutic intervention.

This study aimed to investigate factors (including serum 25-hydroxyvitamin D levels) associated with muscle mass loss after 1 year of assessment in order to identify patients who are more likely to have muscle mass loss.

## Materials and methods

### Patients

This retrospective study enrolled a total of 552 patients with chronic liver disease whose skeletal muscle mass was measured using the BIA method at Nippon Medical School Chiba Hokusoh Hospital between January 2018 and January 2022. Data was collected from June 2022 to November 2022 in six-month periods, and the data was analyzed in December of 2022. Data used in the study were anonymized at the time of entry and managed so that individuals were not immediately identified after data collection. Of the 552 patients, 166 had available data on muscle mass measurements at baseline and at 1 year after the start of the study period. The inclusion criteria were: (1) chronic liver disease diagnosed by laboratory tests and imaging modalities, such as CT and ultrasonography; (2) patient age ≥ 20 years; (3) no supplementation with calcium or vitamin D; and (4) absence of parathyroid dysfunction and liver failure. The exclusion criteria were: (1) pregnancy; (2) pacemaker use; and (3) presence of fluid retention including uncontrollable ascites.

### Laboratory methods

Baseline hematological, biochemical, and clotting test results were collected. Serum 25-hydroxyvitamin D levels that are representative of serum vitamin D levels were measured at the start of the study period using a double-antibody radioimmunoassay kit (SRL, Tokyo, Japan). Cirrhosis was diagnosed based on fibrotic markers, such as platelet count and wisteria floribunda agglutinin positive Mac-2-binding protein, and imaging tests, such as ultrasonography and CT. The body mass index (BMI) was calculated as follows: BMI (kg/m^2^) = body weight (kg) / [height (m)]^2^.

### Definition of skeletal muscle mass loss

BIA was performed using InBody 270 (Biospace, Seoul, Korea) to estimate appendicular skeletal muscle mass, which was calculated as the sum of lean muscle masses of the bilateral upper and lower extremities. The skeletal muscle mass index (SMI), a validated measure for the diagnosis of sarcopenia, was calculated as follows: SMI (kg/m^2^) = appendicular skeletal muscle mass (kg) / [height (m)]^2^. The rate of change from baseline in SMI after 1 year was calculated as follows: ΔSMI (%) = [(1-year SMI − baseline SMI) / baseline SMI] × 100. In the present study, muscle mass loss was defined as ΔSMI ≤ −2%, according to previous reports [8, 10, 21].

### Ethical statement

This study conformed with the ethical guidelines of the Declaration of Helsinki and approved by the Ethics Committee of Nippon Medical Chiba Hokusoh Hospital (approval number 910). Since the study was a retrospective observational study and the data were pseudonymized, informed consent for all patient participation was waived by the Ethics Committee.

### Statistical analyses

Categorical variables are given as numbers. Continuous variables are given as medians and interquartile in parentheses. Univariate and multiple logistic regression analyses were performed to identify independent factors associated with muscle mass loss. A receiver operating characteristic curve was used to determine the optimal cut-off value of serum 25-hydroxyvitamin D to predict muscle mass loss. The Mann–Whitney test was conducted to compare continuous variables with skewed distribution between two groups. Spearman’s rank correlation coefficient was used to evaluate correlations between two groups. Chi-squared test (including adjusted residuals) was used to assess the significance of differences in the distribution of categorical variables among four groups. All statistical analyses were performed using SPSS version 17.0 software (IBM Japan, Tokyo, Japan). The level of significance was set at a p value of < 0.05.

## Results

### Patient characteristics

A total of 166 patients with chronic liver disease 68 (41.0%) males and 98 (59.0%) females; median age [interquartile range (IQR)], 68 (58–74) years were included in this study **(Table 1)**. The etiologies of liver disease were chronic hepatitis C virus (HCV) infection (n = 65), non-alcoholic fatty liver disease (NAFLD; n = 39), chronic hepatitis B virus (HBV) infection (n = 16), alcoholic-related liver disease (ALD; n = 14), primary biliary cholangitis (PBC; n = 14), autoimmune hepatitis (AIH; n = 13), and others (n = 5). Thirteen (7.8%) patients had undergone hepatocellular carcinoma treatment. The treatment of HCC were 2 cases of operation, 9 cases of transcatheter arterial chemoembolization (TACE), 1 case of radiofrequency ablation and 1 case of operation and TACE. The median period from last treatment of HCC to the baseline was 12 months. In other words, this study included patients with a history of HCC, but a certain period of time has elapsed since radical treatment. Thirty-three (19.9%) patients have been receiving BCAA at the start of the study period. Forty-seven (28.3%) patients were diagnosed with cirrhosis. The etiology of 47 liver cirrhosis were HCV (n = 12), HBV (n = 12), ALD (n = 14), AIH (n = 3), PBC (n = 4), NAFLD (n = 11), and others (n = 2). A comparison of etiology between patients with cirrhosis and non-cirrhosis showed that, there were more cirrhosis patients with ALD and HBV (p = 2.90 × 10^−7^, p = 8.31 × 10^−4^), and more non-cirrhosis patients with HCV (p = 8.85 × 10^−5^). The median serum 25-hydroxyvitamin D level was 15.2 (IQR, 11.2–19.3) ng/mL.

**Table 1:** Baseline patient characteristics.

**Factors**	**n = 166**
Age (year)	68 (58–74)
Gender (male/female)	68/98
BMI (kg/m^2^)	24.5 (22.2–28.2)
Etiology of chronic hepatitis HCV/NAFLD/HBV/ALD/PBC/AIH/others	65/39/16/14/14/13/5
History of HCC treatment (yes/no)	13/153
Stage of HCC (I/II/III/IV)	1/11/1/0
Treatment of HCC Operation/TACE/RFA/Operation and TACE	2/9/1/1
Period from the last treatment of HCC (months)	12 (6–15)
BCAA treatment (yes/no)	33/133
LC (yes/no)	47/119
Leukocytes (/mm^3^)	5200 (4248–6300)
Hemoglobin (g/dL)	13.3 (12.4–14.4)
Platelets (×10^3^/mm^3^)	172 (127–219)
AST (U/L)	28 (23–46)
ALT (U/L)	22 (17–40)
γ-GTP (U/L)	31 (20–64)
Total bilirubin (mg/dL)	0.7 (0.5–0.9)
Serum albumin (g/dL)	4.2 (3.9–4.3)
Total cholesterol (mg/dl)	192 (133–225)
Serum creatinine (mg/dL)	0.72 (0.59–0.89)
Prothrombin time (%)	98.5 (86.1–100.0)
WFA^+^-M2BP (C.O.I)	1.15 (0.72–2.48)
Serum 25-hydroxyvitamin D (ng/mL)	15.2 (11.2–19.3)
SMI (kg/m^2^)	6.8 (5.9–7.8)
Muscle mass loss (yes/no)	51/115

Categorical variables are given as numbers. Continuous variables are given as medians and interquartile in parentheses. BMI, body mass index; HCV, hepatitis C virus; NAFLD, nonalcoholic fatty liver disease; HBV, hepatitis B virus; ALD, alcoholic-related liver disease; PBC, primary biliary cholangitis; AIH, autoimmune hepatitis; HCC, hepatocellular carcinoma; TACE, transcatheter arterial chemoembolization; RFA, radiofrequency ablation; BCAA, branched-chain amino acid; LC, liver cirrhosis; AST, aspartate aminotransferase; ALT, alanine aminotransferase; γ-GTP, gamma-glutamyl transpeptidase; WFA^+^-M2BP, Wisteria floribunda agglutinin positive Mac-2-binding protein; COI, cut-off index; SMI, skeletal muscle mass index.

### Changes in SMI for 1 year

**Fig 1:**
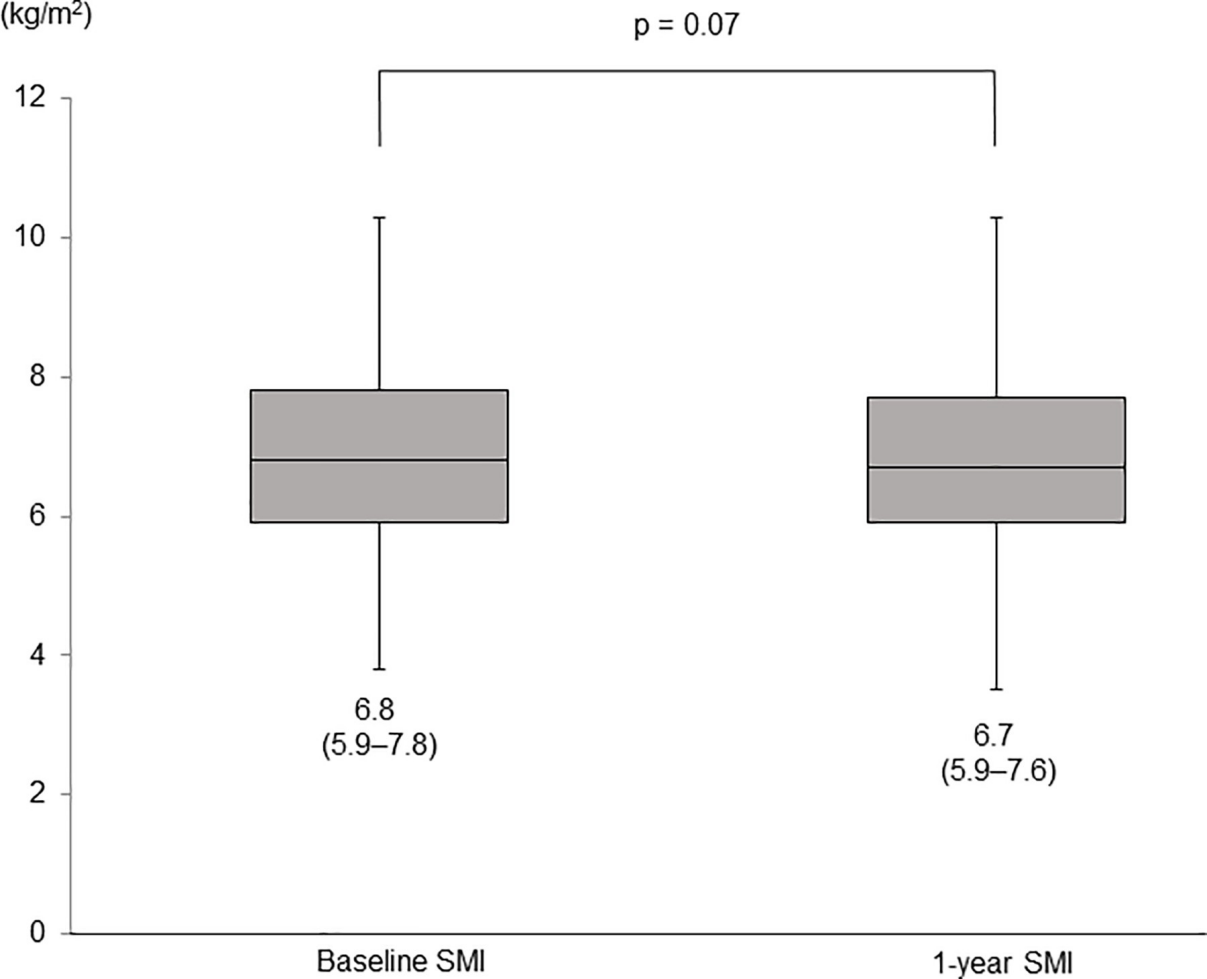
Changes in SMI after one year from baseline. Numbers in parentheses indicate IQR. The ends of the whiskers represent the lowest datum within 1.5 IQR of the lower quartile, and the highest datum within 1.5 IQR of the upper quartile. SMI, skeletal muscle mass index; IQR, interquartile range.

**Fig 2:**
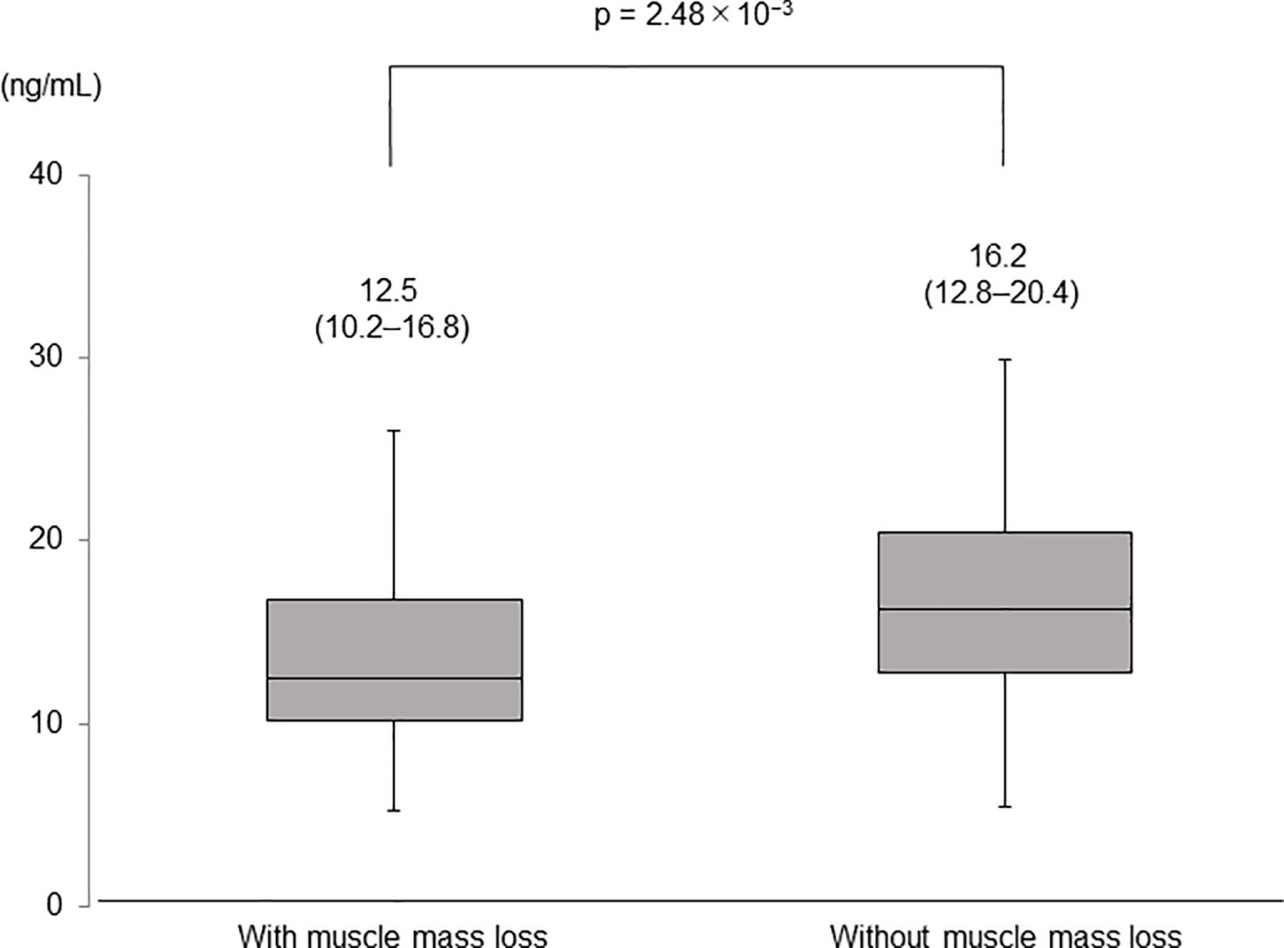
Serum 25-hydroxyvitamin D levels in patients with and without muscle mass loss. Numbers in parentheses indicate IQR. The ends of the whiskers represent the lowest datum within 1.5 IQR of the lower quartile, and the highest datum within 1.5 IQR of the upper quartile. IQR, interquartile range.

In all patients, the median SMI values were 6.8 (IQR, 5.9–7.8) kg/m^2^ at baseline and 6.7 (IQR, 5.9–7.6) kg/m^2^ at the 1-year assessment (p = 0.07, **Fig 1**). Muscle mass loss was found in 51 patients (30.7%) at the 1-year assessment. There were no significant differences in background except for serum 25-hydroxyvitamin D levels between patients with and without muscle mass loss (**Table 2**). The median serum 25-hydroxyvitamin D levels at baseline were 12.5 (IQR, 10.2–16.8) and 16.2 (IQR, 12.8–20.4) ng/mL in patients with and without muscle mass loss, respectively, indicating that the baseline levels were significantly lower in patients with muscle mass loss than in those without (p = 2.48 × 10^−3^, **Fig 2**).

**Table 2:** Baseline patient characteristics with muscle mass loss or without muscle mass loss.

Factors	With muscle mass loss (n = 51)	Without muscle mass loss (n = 115)	P value
Age (year)	69 (60–76)	67 (59–73)	0.35
Gender (male/female)	18/33	50/65	0.39
BMI (kg/m^2^)	25.9 (22.7–29.3)	24.2 (22.0–27.2)	0.06
Etiology of chronic hepatitis HCV/NAFLD/HBV/ALD/PBC/AIH/others	19/19/4/4/3/2/0	46/20/12/10/11/11/5	0.11
History of HCC treatment (yes/no)	5/46	8/107	0.54
BCAA treatment (yes/no)	13/38	20/95	0.29
LC (yes/no)	16/35	31/84	0.46
Leukocytes (/mm^3^)	5020 (4135–6495)	5200 (4290–6215)	0.88
Hemoglobin (g/dL)	13.3 (12.0–14.5)	13.4 (12.6–14.4)	0.42
Platelets (×10^3^/mm^3^)	163 (102–214)	171 (140–224)	0.30
AST (U/L)	30 (24–47)	28 (23–45)	0.63
ALT (U/L)	23 (17–47)	22 (17–37)	0.65
γ-GTP (U/L)	43 (23–7)	32 (20–60)	0.31
Total bilirubin (mg/dL)	0.7 (0.6–1.1)	0.7 (0.5–0.9)	0.33
Serum albumin (g/dL)	4.2 (3.9–4.4)	4.2 (4.0–4.3)	0.89
Total cholesterol (mg/dl)	190 (171–208)	192 (169–212)	0.60
Serum creatinine (mg/dL)	0.75 (0.61–0.89)	0.69 (0.59–0.89)	0.53
Prothrombin time (%)	90.0 (76.9–100.0)	99.8 (86.7–100.0)	0.14
WFA^+^-M2BP (C.O.I)	1.10 (0.88–2.62)	1.10 (0.71–2.22)	0.95
Serum 25-hydroxyvitamin D (ng/mL)	12.5 (10.2–16.8)	16.2 (12.8–20.4)	2.48×10^−3^

Categorical variables are given as numbers. Continuous variables are given as medians and interquartile in parentheses. BMI, body mass index; HCV, hepatitis C virus; NAFLD, nonalcoholic fatty liver disease; HBV, hepatitis B virus; ALD, alcoholic-related liver disease; PBC, primary biliary cholangitis; AIH, autoimmune hepatitis; HCC, hepatocellular carcinoma; BCAA, branched-chain amino acid; LC, liver cirrhosis; AST, aspartate aminotransferase; ALT, alanine aminotransferase; γ-GTP, gamma-glutamyl transpeptidase; WFA^+^-M2BP, Wisteria floribunda agglutinin positive Mac-2-binding protein; COI, cut-off index.

**Fig 3:**
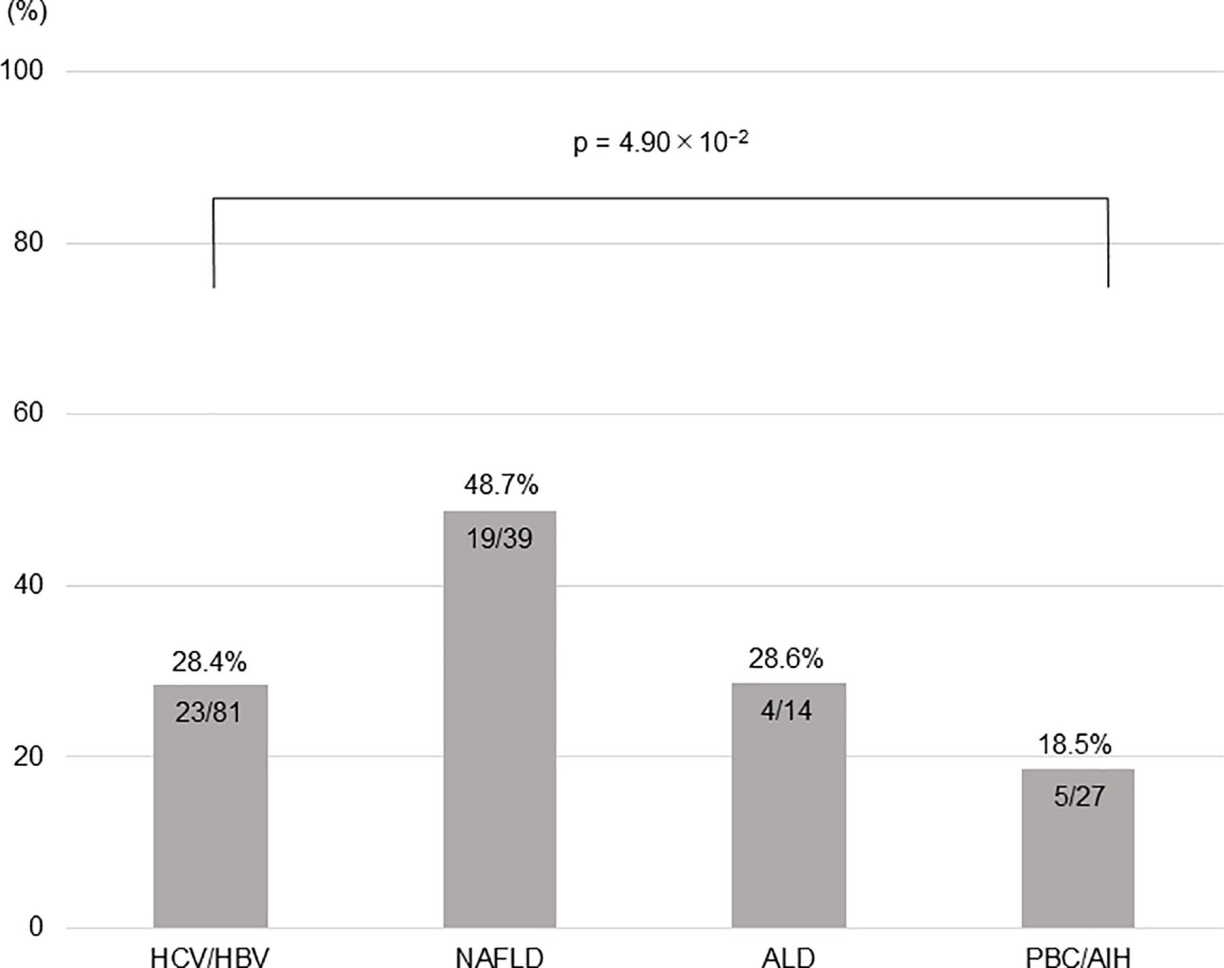
Incidence of muscle mass loss after 1 year according to etiology. HCV, hepatitis C virus; HBV, hepatitis B virus; NAFLD, nonalcoholic fatty liver disease; ALD, alcoholic-related liver disease; PBC, primary biliary cholangitis; AIH, autoimmune hepatitis.

The incidence of muscle mass loss was 28.4% (23/81) in patients with HBV/HCV, 48.7% (19/39) in those with NAFLD, 28.6% (4/14) in those with ALD, and 18.5% (5/27) in those with PBC/AIH. The patients with NAFLD showed the highest incidence of muscle mass loss (p = 4.90 × 10^−2^, adjusted residual = |2.6|, **Fig 3**).

### Muscle mass loss in patients with cirrhosis and non-cirrhosis

The incidences of muscle mass loss in patient with cirrhosis were 36.2% (17/47) and in patients with non-cirrhosis was 28.6% (34/119), respectively. There were no significant differences in muscle mass loss between patients with cirrhosis and non-cirrhosis (p = 0.36, **S1 Fig**). The median ΔSMI was 0% (IQR, −3.00% to 0%) in patients with cirrhosis, and −1.27% (IQR, −1.79% to 0%) in patients with non-cirrhosis, respectively (p = 0.36, **S2 Fig**).

### Factors associated with muscle mass loss after 1 year

On univariate analysis, low serum 25-hydroxyvitamin D level (OR, 1.18; 95% CI, 1.04–1.18; p = 8.20 × 10^−4^) was significantly associated with muscle mass loss. On multivariate analysis, low serum 25-hydroxyvitamin D level (OR, 1.10; 95% CI, 1.03–1.18; p = 4.31 × 10^−3^) remained a significant and independent factor associated with muscle mass loss (**Table 3**).

**Table 3:** Univariate and multiple logistic regression analyses for factors associated with muscle mass loss after 1 year from baseline. In the multivariate analysis, all factors were adjusted and analyzed.

Factors	Category	Univariate			Multivariate		
		OR	95%CI	P value	OR	95%CI	P value
Age	By 1 year old up	1.01	0.98–1.04	0.60			
Gender	Female	1.54	0.80–2.97	0.19			
Etiology of chronic hepatitis	NAFLD	1.64	0.78–3.46	0.19			
BMI	Less than 22 kg/m^2^	0.68	0.31–1.50	0.34			
Hemoglobin	By 0.1 g/dl down	1.16	0.97–1.39	0.10			
Albumin	By 0.1 g/dL down	1.01	0.60–2.46	0.58			
History of HCC treatment	Presence	1.32	0.44–4.00	0.62			
BCAA treatment	Presence	1.15	0.51–2.59	0.74			
Liver cirrhosis	Presence	1.40	0.71–2.81	0.33			
Serum 25-hydroxyvitamin D	By 0.1 ng/mL down	1.18	1.04–1.18	8.20×10^−4^	1.10	1.03–1.18	4.31×10^−3^

*OR, odds ratio; CI, confidence interval; NAFLD, nonalcoholic fatty liver disease; body mass index; HCC, hepatocellular carcinoma; BCAA, branched-chain amino acid

Optimal cut-off value of serum 25-hydroxyvitamin D for muscle mass loss after 1 year and ΔSMI according to serum 25-hydroxyvitamin D levels

**Fig 4:**
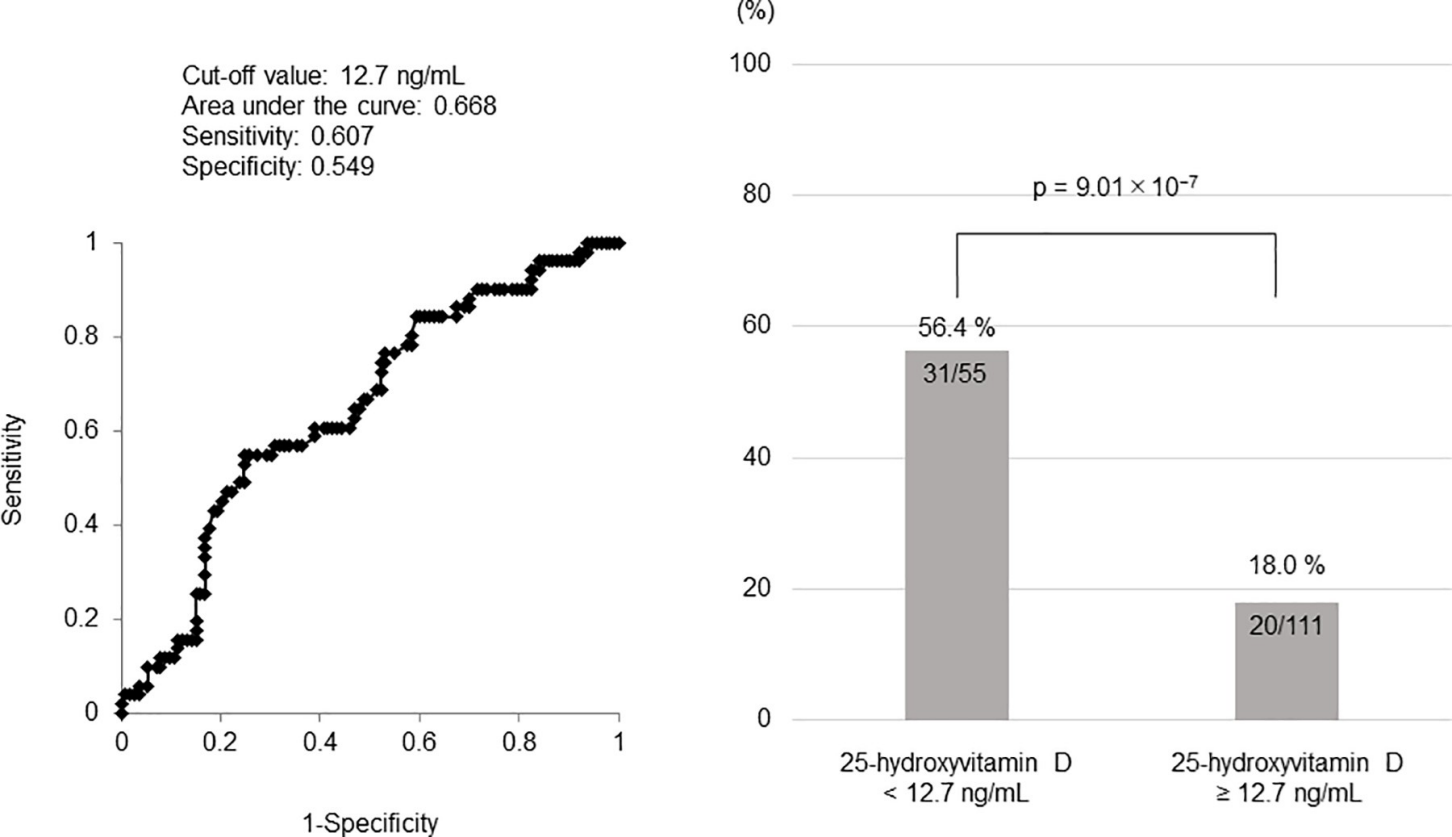
The cut-off value for serum 25-hydroxyvitamin D level to predict muscle mass loss. The incidence of muscle mass loss in patients with serum 25-hydroxyvitamin D level ≥ 12.7 and < 12.7 ng/mL (cut-off value, 12.7 ng/mL).

**Fig 5:**
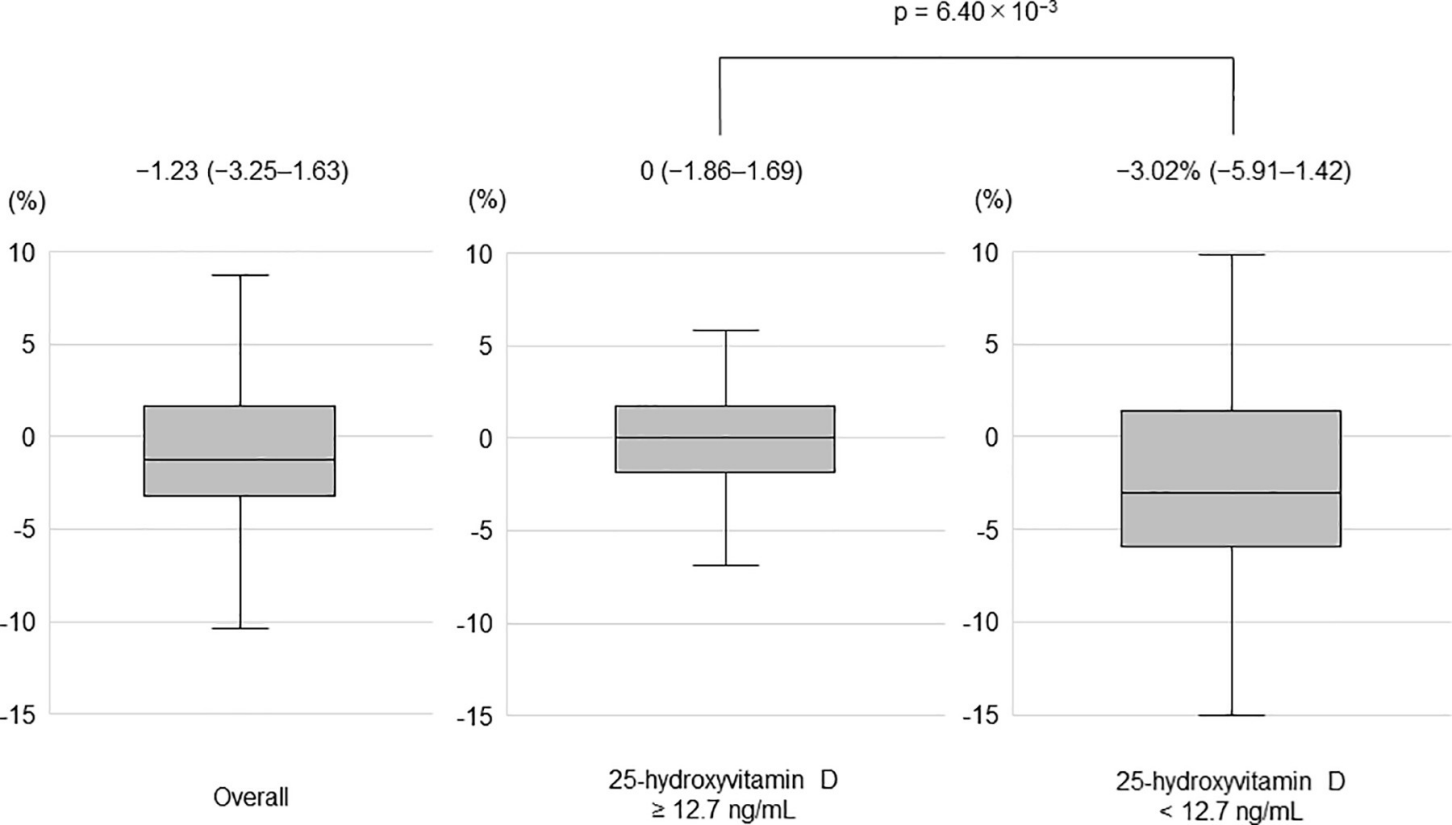
The rate of change in SMI from baseline after 1 year in all patients and those with serum 25-hydroxyvitamin D level ≥ 12.7 and < 12.7 ng/mL. Numbers in parentheses indicate IQR. The ends of the whiskers represent the lowest datum within 1.5 IQR of the lower quartile, and the highest datum within 1.5 IQR of the upper quartile. IQR, interquartile range.

The optimal cut-off value for serum 25-hydroxyvitamin D was 12.7 ng/mL (area under the curve, 0.668; sensitivity, 60.7%; specificity, 54.9%). Muscle mass loss was found in 56.4% (31/55) and 18.0% (20/111) of patients with serum 25-hydroxyvitamin D level < 12.7 and ≥ 12.7 ng/mL, respectively (p = 9.01 × 10^−7^, **Fig 4**). Patients with serum 25-hydroxyvitamin D level < 12.7 ng/mL were more likely to have BCAA treatment and complicated cirrhosis (p = 6.42 × 10^−5^, p = 3.19 × 10^−3^). Patients with serum 25-hydroxyvitamin D level < 12.7 ng/mL were significantly lower hemoglobin (p = 7.90 × 10^−3^), platelet (p = 9.30 × 10^−3^), and prothrombin time (p = 1.43 × 10^−3^) and total cholesterol (p = 4.71 × 10^−3^) than patients with serum 25-hydroxyvitamin D level ≥ 12.7 ng/mL. On the other hand, significantly high gamma-glutamyl transpeptidase and wisteria floribunda agglutinin positive Mac-2-binding protein than patients with serum 25-hydroxyvitamin D level ≥ 12.7 ng/mL (p = 1.82 × 10^−2^, p = 2.43 × 10^−3^, **Table 4**). The median ΔSMI was −1.23% (IQR, −3.25% to 1.63%) in the overall cohort, and 0% (IQR, −1.86% to 1.69%) and −3.02% (IQR, −5.91% to 1.42%) in patients with serum 25-hydroxyvitamin D level ≥ 12.7 and < 12.7 ng/mL, respectively (p = 6.40 × 10^−3^, **Fig 5**).

**Table 4:** Baseline patient characteristics according to serum 25-hydroxyvitamin D level.

Factors	serum 25-hydroxyvitamin D level < 12.7 (n = 55)	serum 25-hydroxyvitamin D level ≥ 12.7 (n = 111)	P value
Age (year)	68 (58–75)	67 (59–73)	0.96
Gender (male/female)	19/36	49/62	0.25
BMI (kg/m^2^)	25.6 (21.6–30.3)	24.4 (22.3–27.1)	0.30
Etiology of chronic hepatitis HCV/NAFLD/HBV/ALD/PBC/AIH/others	16/18/3/9/6/3/0	49/21/13/5/8/10/5	1.17×10^−2^
History of HCC treatment (yes/no)	7/48	6/105	0.13
BCAA treatment (yes/no)	21/34	12/99	6.42×10^−5^
LC (yes/no)	24/31	23/88	3.19×10^−3^
Leukocytes (/mm^3^)	4680 (4020–6315)	5300 (4410–6270)	0.24
Hemoglobin (g/dL)	12.6 (11.4–14.1)	13.5 (12.9–14.5)	7.90×10^−3^
Platelets (×10^3^/mm^3^)	146 (92–203)	178 (147–224)	9.30×10^−3^
AST (U/L)	32 (25–49)	27 (23–41)	0.09
ALT (U/L)	25 (18–42)	21 (17–39)	0.54
γ-GTP (U/L)	47 (24–94)	29 (19–54)	1.82×10^−2^
Total bilirubin (mg/dL)	0.7 (0.5–0.9)	0.8 (0.6–1.2)	0.09
Serum albumin (g/dL)	4.0 (3.6–4.3)	4.2 (4.0–4.4)	3.83×10^−4^
Total cholesterol (mg/dl)	180 (149–204)	192 (177–215)	4.71×10^−3^
Serum creatinine (mg/dL)	0.69 (0.59–0.90)	0.74 (0.59–0.89)	0.50
Prothrombin time (%)	86.3 (70.8–100.0)	100.0 (87.9–110.0)	1.43×10^−3^
WFA^+^-M2BP (C.O.I)	1.79 (0.91–5.09)	1.00 (0.71–1.49)	2.43×10^−3^

Categorical variables are given as numbers. Continuous variables are given as medians and interquartile in parentheses. BMI, body mass index; HCV, hepatitis C virus; NAFLD, nonalcoholic fatty liver disease; HBV, hepatitis B virus; ALD, alcoholic-related liver disease; PBC, primary biliary cholangitis; AIH, autoimmune hepatitis; HCC, hepatocellular carcinoma; BCAA, branched-chain amino acid; LC, liver cirrhosis; AST, aspartate aminotransferase; ALT, alanine aminotransferase; γ-GTP, gamma-glutamyl transpeptidase; WFA^+^-M2BP, Wisteria floribunda agglutinin positive Mac-2-binding protein; COI, cut-off index.

There was no correlation between baseline serum 25-hydroxyvitamin D level and ΔSMI (*r* = −0.072, p = 0.36).

### Incidence of muscle mass loss according to serum 25-hydroxyvitamin D levels in each etiology

**Fig 6:**
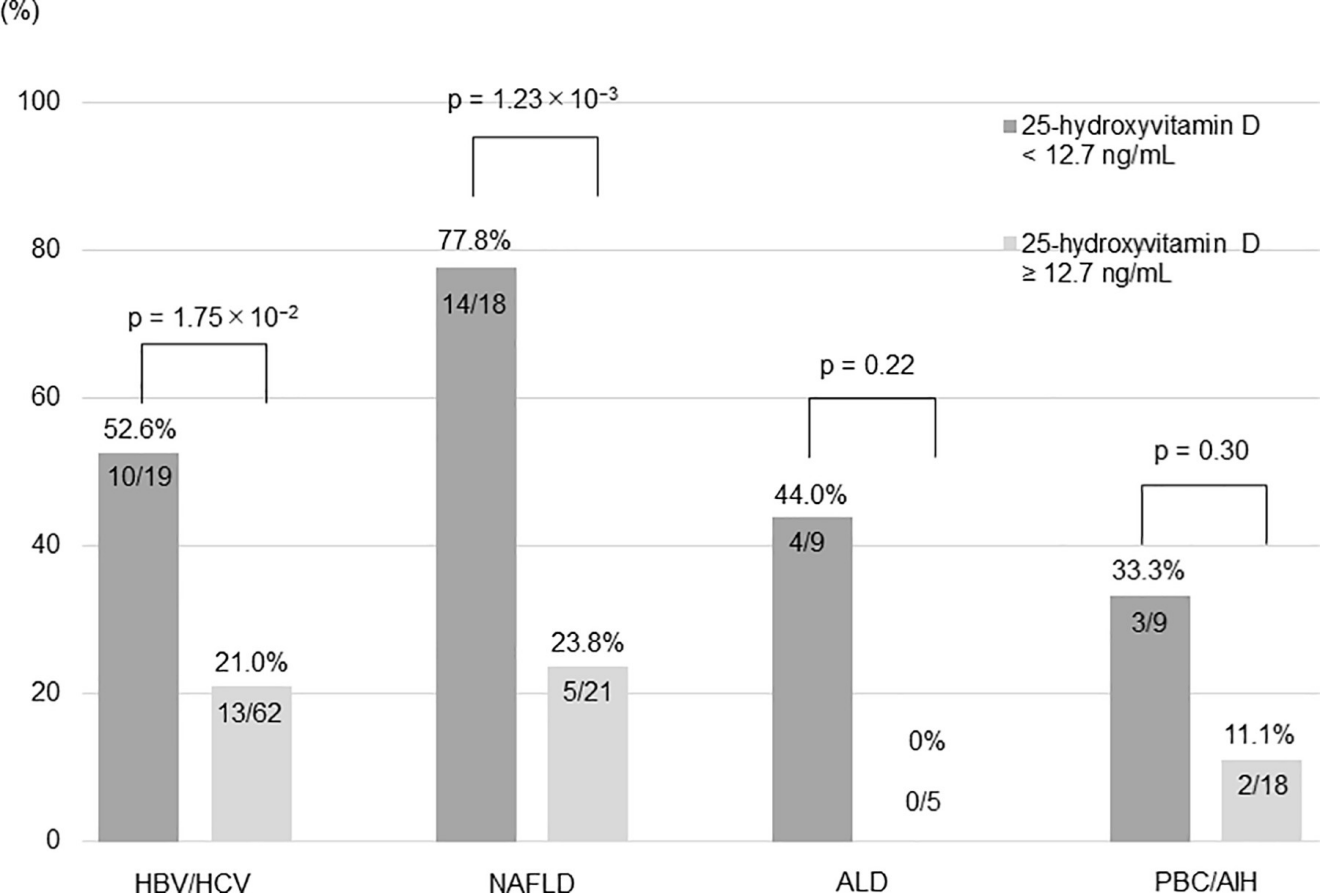
Incidence of muscle mass loss after 1 year according to serum 25-hydroxyvitamin D levels in each etiology. HCV, hepatitis C virus; HBV, hepatitis B virus; NAFLD, nonalcoholic fatty liver disease; ALD, alcoholic-related liver disease; PBC, primary biliary cholangitis; AIH, autoimmune hepatitis.

The incidence of muscle mass loss at serum 25-hydroxyvitamin D levels < 12.7 ng/mL was observed in 52.6% (10/19) in patients with HBV or HCV, 77.8% (14/18) in patients with NAFLD, 44.0% (4/9) in patients with ALD, and 33.3% (3/9) in patients with PBC or AIH. In patients with HBV/HCV and NAFLD, the incidence of muscle mass loss was significantly higher in patients with serum 25-hydroxyvitamin D level < 12.7 ng/mL than in those with ≥ 12.7 ng/mL (52.6% vs. 21.0%; p = 1.75 × 10^−2^ and 77.8% vs. 23.8%; p = 1.23 × 10^−3^, **Fig 6**). In patients with ALD and PBC/AIH, the incidence of muscle mass loss was higher in patients with serum 25-hydroxyvitamin D level < 12.7 ng/mL than in those with ≥ 12.7 ng/mL, though not statistically significant.

## Discussion

A 3-year follow-up study of community-dwelling older adults (n = 1,008; age ≥ 65 years) reported that individuals with low baseline 25-hydroxyvitamin D levels (< 10 ng/mL) were 2.14 times more likely to experience a loss of muscle mass (< −3%) than those with high levels (> 20 ng/mL), suggesting that vitamin D-deficient status could progress sarcopenia regardless of advancing age [16]. In Chinese community-dwelling older adults, the decrease rates in appendicular skeletal mass were reported to be −0.66% and −0.94% after 2 years and −1.60% and −2.02% after 4 years in men and women, respectively [21]. In the present study, it was −1.23% after 1 year in the overall cohort, indicating that the annual decrease ratio in muscle mass in patients with chronic liver disease is greater and more progressive than that in the general population. Furthermore, low baseline 25-hydroxyvitamin D levels (< 12.7 ng/mL) were significantly and independently associated with muscle mass loss (≤ −2%), suggesting that chronic liver disease complicated by vitamin D deficiency may accelerate muscle mass loss faster than other factors, such as aging. Early detection of faster muscle mass loss and treatment intervention are required to prevent the development or progression of sarcopenia in patients with chronic liver disease, given that liver cirrhosis can lead to deterioration of general condition and decline in activities of daily living.

Sarcopenia in patients with cirrhosis is caused and exacerbated by multiple factors, such as portal hypertension, pro-inflammatory cytokines, hyperammonemia, administration of loop diuretics, hypotestosteronemia, physical inactivity, elevated hepatic gluconeogenesis, impaired insulin/IGF-1 signaling, and excessive alcohol consumption [22]. We previously reported that low vitamin D levels are associated with sarcopenia in patients with chronic liver disease [15]. Vitamin D deficiency can cause atrophy of type II (fast-twitch muscle fibers) fibers and fatty infiltration of skeletal muscle, which are involved in sarcopenia [23]. Myostatin is a cytokine belonging to the transforming growth factor beta 1 family and a negative regulator of muscle hypertrophy; its quantity increases in patients with cirrhosis [24], thereby inhibiting skeletal myogenesis. Intriguingly, it has been found to increase in mice with vitamin D deficiency [25]. We reported that vitamin D supplements maintained and improved skeletal muscle mass in patients with cirrhosis [10]. The European Association for the Study of the liver guidelines recommend that patients with low vitamin D levels should take oral vitamin D supplementation to maintain serum vitamin D levels at > 30 ng/mL [6]. In the present study, 5 patients with serum 25-hydroxyvitamin D level > 30 ng/mL did not show muscle mass loss for 1 year. Therefore, patients with low vitamin D levels should be carefully monitored or treated despite the absence of sarcopenia.

In general, vitamin D levels are lower in patients with NAFLD [26–29]. Furthermore, it has been reported that low serum vitamin D levels are associated with sarcopenia, NAFLD, and sarcopenia in NAFLD [30]. In the present study, patients with NAFLD had the highest incidence of muscle mass loss among patients with different etiologies. Furthermore, 76.5% of those with a serum 25-hydroxyvitamin D level < 12.7 ng/mL had a significantly higher incidence of muscle mass loss. Although muscle mass loss and a low serum 25-hydroxyvitamin D level were well-associated, no correlation was found between serum 25-hydroxyvitamin D level and ΔSMI. This result is considered to be due to the complexity of the relationship between muscle mass loss and the amount of activity in daily life. As body fat mass increases, vitamin D is sequestered in adipose tissue and serum vitamin D concentrations decrease [31]. In the present study, the mechanisms of the incidence of muscle mass loss were higher in patients with NAFLD with low vitamin D levels then other etiologies were discussed as follows. First, patients with NAFLD with increased body fat mass are more likely to have muscle mass loss as result of decreased vitamin D levels. Second, patients with NAFLD may have muscle mass loss due to decreased exercise and activity in their daily lives. Retention of vitamin D level while reducing body fat mass might prevent loss of muscle mass. These findings suggest that patients with NAFLD, especially those with low vitamin D levels, may be predisposed to muscle mass loss and requires more aggressive preventive intervention. Given that vitamin D is biosynthesized in the skin by sunlight [32], outdoor exercise therapy that allows exposure to sunlight may be effective in reducing visceral fat and increasing vitamin D, especially in patients with NAFLD with low vitamin D levels.

The present study has several limitations. First, the number of patients was small and from a single institution. Second, muscle mass was measured by the BIA method, patients with fluid retention were excluded because the BIA method may not accurately assess muscle mass [33]. Therefore, cirrhotic patients with fluid retention, which has a high complication rate of sarcopenia, were not included in this study. To address this issue, combination of other methods unaffected by fluid retention, such as the CT-based measuring method, is required. Third, the observation period in this study was not long enough to determine the long-term relationship between serum vitamin D levels and muscle mass loss. Forth, this study lacks information on physical activities that may affect muscle mass. Fifth, only the skeletal muscle mass was measured in this study, excluding grip strength, which is included in the diagnosis criteria of sarcopenia [5] and may influence patient prognosis [34]. The study included patients with chronic liver disease of various etiologies, but relatively few patients with AIH or PBC. Therefore, it is not clear whether the results of this study are reflected in patients with all etiologies. It is necessary to conduct similar analyses in patients with such backgrounds in the future to clarify the results. Also, it is still unclear that early muscle mass loss is associated with sarcopenia complications. Long-term studies with a larger cohort and those that include the assessment of grip strength are needed.

## Conclusion

Low vitamin D levels were significantly and independently associated with muscle mass loss for 1 year in patients with chronic liver disease. The incidence of muscle mass loss was higher especially in patients with NAFLD with low vitamin D, highlighting the need for preventive intervention in this population.

## Supporting information

S1 Fig Incidence of muscle mass loss after 1 year in patients with cirrhosis and non-cirrhosis. (TIF)

S2 Fig The rate of change in SMI from baseline after 1 year in patients with cirrhosis and non-cirrhosis. Numbers in parentheses indicate IQR. The ends of the whiskers represent the lowest datum within 1.5 IQR of the lower quartile, and the highest datum within 1.5 IQR of the upper quartile. IQR, interquartile range.
(TIF)

S1 Data (XLSX)
