# Supplementary figures and images for: Low vitamin D levels accelerates muscle mass loss in patients with chronic liver disease
Source: PLoS One. 2024 Mar 26;19(3):e0299313. doi: 10.1371/journal.pone.0299313 (PMC10965050; doi:10.1371/journal.pone.0299313)

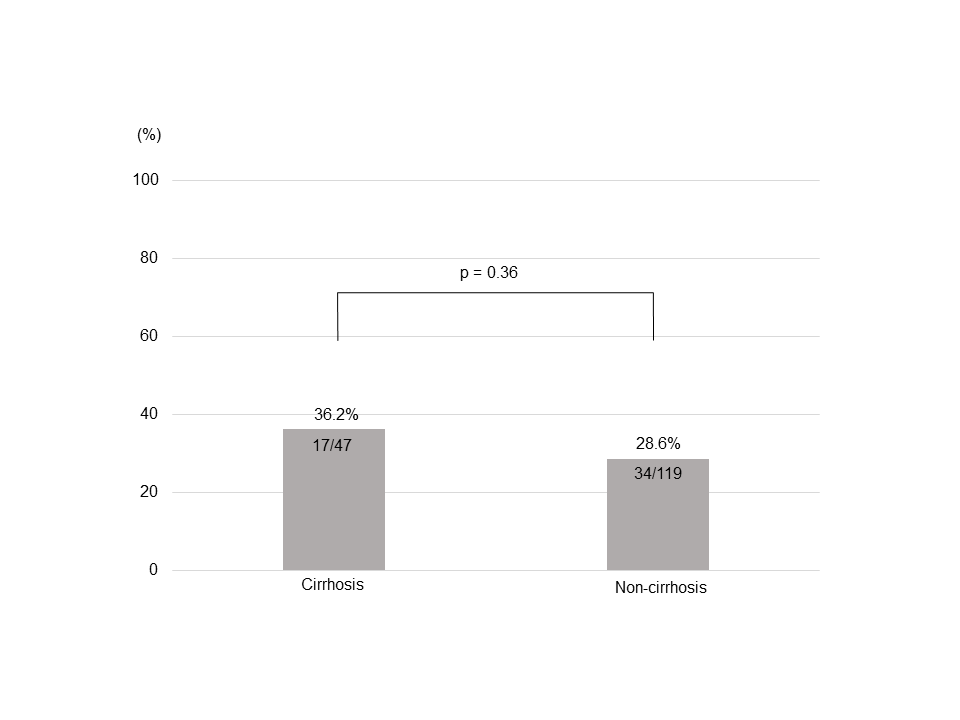

Supplement: S1 Fig — (TIF) [file pone.0299313.s001.tif]

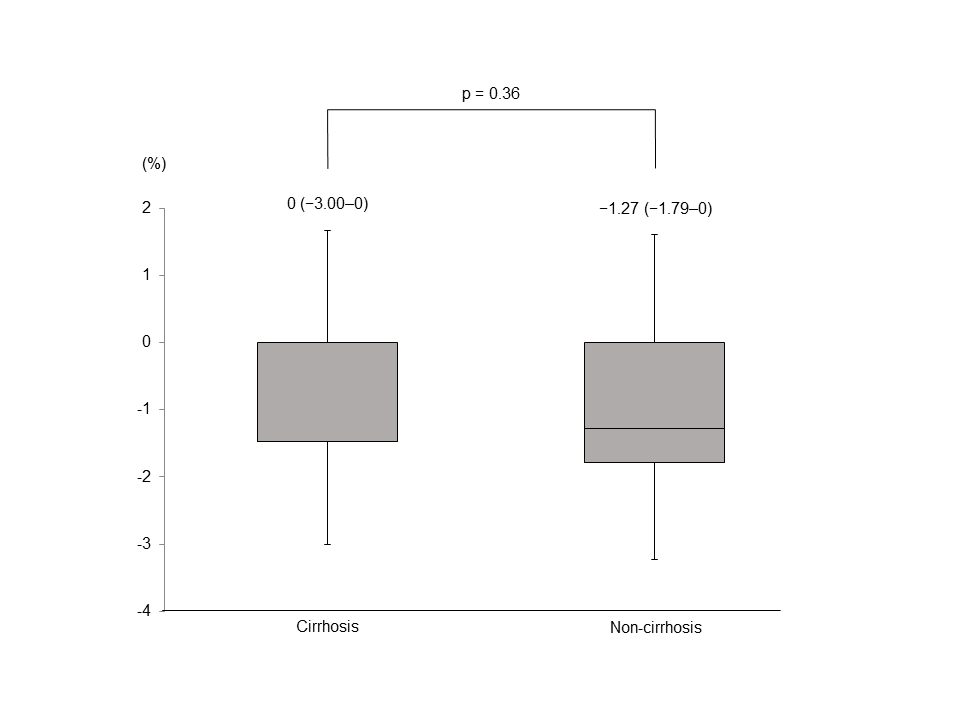

Supplement: S2 Fig — Numbers in parentheses indicate IQR. The ends of the whiskers represent the lowest datum within 1.5 IQR of the lower quartile, and the highest datum within 1.5 IQR of the upper quartile. IQR, interquartile range. (TIF) [file pone.0299313.s002.tif]
